# Sharing research data

**DOI:** 10.1177/0309364620915020

**Published:** 2020-04-21

**Authors:** Nerrolyn Ramstrand, Stefania Fatone, Michael P Dillon, Brian J Hafner

**Affiliations:** 1 Jönköping University, Jönköping, Sweden; 2 Northwestern University, Chicago, IL, USA; 3 La Trobe University, Melbourne, VIC, Australia; 4 University of Washington, Seattle, WA, USA

In our October 2019 editorial, we focused on research best practice by encouraging authors to register clinical trials and systematic reviews in public registers, as well as utilising reporting guidelines when writing scholarly articles.^1^ In this editorial, we continue our discussion of ways to promote quality and transparency in research by encouraging authors to share their research data.

Data sharing refers to the practice of saving research data (e.g. measurements, observations, and transcripts) and metadata (i.e., information about the data that facilitate understanding of different aspects of the data) in publicly available locations so that they can be accessed and used by others. There are a number of ways that authors can share their research data. One way is to submit supplemental material, related to a specific publication, with the manuscript itself. The supplemental data file will be hosted by the publisher, or a third party, and linked to the published article. Authors can also host their data on a public (e.g. institutional) website or submit their data to a public data repository (e.g. Zenodo,^2^ Figshare,^3^ UK Data Service^4^).

While each of these data sharing strategies helps to increase access to research data, hosting data in a public repository is increasingly recommended by publishers and granting agencies for several reasons. First, data hosted in a public repository are available to everyone. Data hosted by a publisher, for example, may be restricted to only select individuals (e.g. journal subscribers). Second, data hosted in public repositories are indexed and searchable, meaning data sets can be browsed and easily identified. Third, public repositories may allow authors to select different types of licences that restrict how the data are accessed, used, acknowledged, or redistributed. Finally, data hosted in a repository are often assigned a digital object identifier (DOI), which can be used to cite the work in perpetuity. Collectively, these benefits make public repositories an ideal option for authors wishing to share their research data.

While much progress is needed before data sharing becomes standard practice, a number of international scientific bodies have already taken steps to support public access to research data. The European Commission, the United States Office of Science and Technology Policy, and the Global Research Council all mandate open data from their grant recipients.^5^

## Why should I share my research data?

Research data continue to be a valuable commodity even when the specific project for which it was generated has ended. By making data available to other investigators, researchers create opportunities for new scientific initiatives. Sharing also facilitates combining the data into larger, more robust data sets that can then be used for other purposes (e.g. meta-analyses). Research data sharing also has ethical benefits in that it reduces the need for duplicate data collection, limits unnecessary risk to participants, and maximises potential for research output from the initial investment, which is often funded using public money. By making their data sets available to the public, researchers are also taking steps to improve the transparency and reproducibility of their research findings by allowing others to verify their results.

## Documenting your data

In order to ensure that others can access, understand and use repository data appropriately, researchers must undertake careful planning from the outset of the project. A *data management plan*, written in the early phase of a project, and updated to include a history of changes throughout the project, can be particularly useful in helping researchers organise, structure, and manage their data so that it can be efficiently and effectively shared at the end of a project. A data management plan should detail the data that researchers expect to collect during a project, how they intend to manage the data, how they will describe the data, and how they will store the data. It should also detail how and what mechanisms will be used to preserve and share the data upon completion of the project. There are many good resources available to assist researchers in developing data management plans. Examples include the United States Geographical Survey (USGS) data management plan checklist,^6^ and the UK data service data management webpage.^7^

When generating a data management plan and preparing data for registration in a freely accessible repository, we recommend that authors abide by the FAIR principles.^8^ The FAIR principles were developed to improve the Findability, Accessibility, Interoperability, and Reuse of data. The principles are presented in Table 1 and provide a useful framework for researchers to prepare their data in a way that will maximise use and reuse.

**Table 1.** FAIR guiding principles for data management and stewardship.

Findability	Accessibility	Interoperability	Reuse
F1. (Meta)data are assigned a unique and permanent identifier.	A1. (Meta)data are retrievable by their identifier using a standardised communications protocol. A1.1 The protocol is open, free and universally implementable. A1.2 The protocol allows for an authentication and authorisation procedure, where necessary.	I1. (Meta)data use a formal, accessible, shared and broadly applicable language for knowledge representation.	R1. Meta(data) are richly described with accurate and relevant attributes. R1.1. (Meta)data are released with a clear and accessible data usage licence. R1.2. The origin of (Meta)data are clear. R1.3. (Meta)data meet domain-relevant community standards.
F2. Data are described with rich metadata (defined by R1).	A2. Metadata are accessible, even when the data are no longer available.	I2. (Meta)data use vocabularies that follow FAIR principles.	
F3. Metadata clearly and explicitly include the identifier of the data they describe.		I3. (Meta)data include qualified references to other (meta)data.	
F4. (Meta)data are registered or indexed in a searchable resource.			

*Source*: Adapted from https://www.go-fair.org/fair-principles/.

FAIR: Findability, Accessibility, Interoperability, and Reuse of data.

## Challenges associated with data sharing

There are a number of challenges associated with sharing data, and it is important to consider these issues before making the decision to share data in an open repository.

A common concern noted by academic researchers, who often spend years on a project, is the possibility that sharing their data too early will lead to their competitors gaining recognition for work they have initiated. There is also a fear by some investigators that their data will be misused or misinterpreted.^9^ An example of this could be the failure by other researchers to consider the environment in which the original data was generated or the nature of the patient group under investigation.^9^ A third concern is protection and control over research data when it is left in the hands of a third party (e.g. owners of the repository). While there are no clear-cut solutions to these issues, work is currently being undertaken to expand sharing of data while retaining the trust of those who store and retrieve it. One example is the Data Trust Project, initiated by the Open Data Institute in 2018 with the aim of developing legal structures to provide independent stewardship of data.^10^

In addition to concerns that have obvious consequences for individual researchers, there are also ethical issues to be addressed in relation to data sharing. It is important to realise that not all data can, or should, be made publicly available. Prior to making data available, we suggest that authors consider the following:

- Does the data set contain private or sensitive information?
- Is it possible to de-identify data so the individual participants cannot be distinguished and such that it cannot be re-linked with other data to create identifiable information?
- Can confidential information be appropriately managed?
- Is the quality of the data acceptable for reuse?
- Have participants consented to future reuse of their data?
- Are participants aware of how and where their data will be stored and maintained?

The extent to which authors can address these issues will depend on their initial planning and the nature of the research data they are collecting. For authors who wish to gain more insight into these issues, we recommend reading Michelle Meyer’s *Practical Tips for Ethical Data Sharing*.^11^

When selecting a data repository, we recommend that authors refer to the checklist developed by Whyte,^12^ which encourages researchers to address the following questions:

- Is a reputable repository available?
- Will it take the data you want to deposit?
- Will it be safe in legal terms?
- Will the repository sustain the data value?
- Will it support analysis and track data usage?

## How are data stored and shared at *Prosthetics and Orthotics International*?

*Prosthetics and Orthotics International* authors who wish to share their research data can choose among several options at the time of submission. Authors can submit their research data alongside their other materials. In this situation, if the manuscript is published, SAGE will automatically archive data in an open data sharing repository called Figshare.^13^ Alternatively, authors can choose to host their data in another publicly accessible location (e.g. website or research data repository), and then cite the location of the data in the manuscript under the heading *Data Accessibility Statement*.

## Conclusion

Responsible data sharing offers considerable benefits to researchers, funding agencies and the public. It promotes transparency in research, facilitates novel scientific inquiry, avoids duplication of effort and maximises benefits of the original research investment. We believe that *Prosthetics and Orthotics International* can play an important role in promoting and supporting data sharing initiatives. When appropriate, and with careful attention to ethical principles, we encourage our authors to share their research data in reputable data repositories.
